# The Women’s Brain Project: an interview with Annemarie Schumacher Dimech on addressing gender in STEM and mental health research

**DOI:** 10.1038/s42003-021-02699-6

**Published:** 2021-10-11

## Abstract

World Mental Health Day and Ada Lovelace Day are just 2 days apart. Whilst we use World Mental Health Day on October 10 to highlight what still needs to be done to address some of the gaps in our understanding of mental health, Ada Lovelace Day on October 12 celebrates Women in STEM. We spoke to Dr. Annemarie Schumacher Dimech—President and co-founder of the Women’s Brain Project—who embodies both days and strives to advance not only our gender-specific understanding of mental health but is also flying the flag for women in STEM careers.

Dr. Annemarie Schumacher Dimech is President of the Women’s Brain Project (WBP) and holds an MSc in Health Psychology from the University of Surrey. In 2010, she obtained her PhD at the University of Bern. Today, she is at the University of Lucerne where she developed and currently heads its program of further education in Palliative Care. Her fascination with the interaction between body and mind motivates her to study physical and socioeconomic factors affecting our mental health and this forms the basis of her work with the WBP, where she contributes to educational and scientific events, publications, and research. Sex and gender differences in our health, including socioeconomic and psychological factors, which affect brain and mental health was Annemarie’s motivation to join forces with Antonella Santuccione Chadha, Maria Teresa Ferretti, and Gautam Maitra to found the WBP. Through the WBP, increasing awareness and sharing knowledge about these differences can lead to changes that will significantly improve women’s health and society’s wellbeing.

**Figure:**
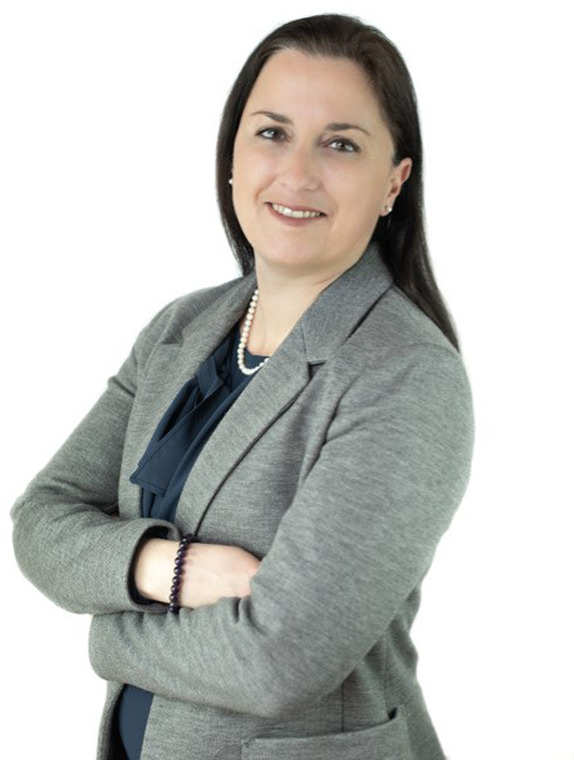
Annemarie

What is the Women’s Brain Project (WBP) and what inspired you to co-found it?

The WBP starting point was a passion about one’s work, personal experiences and a motivation to change things for the better. After 5 years, I look back and am amazed at how this dream “took form and took off” in such a short span of time! We started off as a group of four people brainstorming an idea we felt strongly about. The idea of WBP was conceived by the co-founders Dr. Antonella Santuccione Chadha, Gautam Maitra, Dr. Maria Teresa Ferretti and I, and born in further discussions with the community of researchers, academics, clinicians, and professionals interested in promoting gender-based science for the benefit of humanity.

Today, WBP is a registered non-profit organization with an international team of over 30 people and an advisory board of world-renowned experts. The team is composed of scientists specialized in various disciplines including medicine, neuroscience, psychology, and pharmacy. We collaborate with policymakers, pharmaceutical companies, caregivers, patients and their families as well as other stakeholders. Our aim is to advance a global discussion on gender and sex determinants of vulnerability to brain and mental disease, address sex and gender bias in research, treatment and prevention of brain and mental diseases. We also aim to promote a precision medicine approach to provide better, more tailored and cost-effective care to patients. Most importantly, WBP strives to generate scientific evidence as why the consideration of sex and gender matters in brain and mental disease both in medicine as well as other fields of healthcare.

This may sound over-ambitious for such a “young” organization, however, our achievements in these 4 years speak for themselves. Apart from growing to the team we are now, we have published various peer-reviewed publications as well as many interviews and articles in the general media. WBP has been invited in various events both scientific as well as for the lay public, including the World Economic Forum, and has established collaborations with international organizations and academic institutions. Last but not least, WBP’s flagship event is the International Forum on Women’s Brain and Mental Health. This event’s main goal is to bring together experts and representatives from a wide range of scientific disciplines and various fields of practice contributing to the study and improvement of mental and brain health. The amazing teamwork, considering the team works pro bono on their tasks, and the results achieved in such a short span of time inspires me to continue on this path. Moreover, I find the WBP team members to be a source of inspiration for me where I not only enjoy the scientific exchanges and collaboration on projects but also learn from their experience and expertise. Moreover, the unexpected but very positive reactions from my networks, both private and professional, are also an inspiration to continue on this path. On talking about WBP’s aims, many friends and colleagues shared their personal stories which confirmed the need and importance of WBP’s work.

What hurdles have you faced professionally as a woman in STEM?

I am lucky not to have faced extreme hurdles in the course of my studies and career. However, I do feel a change in my work environment in comparison to when I started my career. As a young researcher, I sometimes felt I was not taken seriously or not considered for particular tasks. I experience this less now, which I think is a result of experience and becoming more self-confident and assertive. I encourage women to believe in their abilities and speak up!

Our work and contributions to STEM will help change perceptions and bias and encourage more girls and women to study and pursue a career in STEM.

Do you have any female role models that have been instrumental to your career?

My first inspiration by a woman in STEM happened in fourth grade when I came across a children’s book about Marie Curie in our school library. I was so fascinated by this woman’s story and her ground-breaking work in diagnostics. I also remember the illustrations of Marie Curie with big skirts and attire of that period, working in her lab, presenting me with a picture showing that being female does not exclude you from being able to work in this field.

I have been lucky to also meet many amazing women in real life who have inspired me in my private and professional life. I am even more lucky to be able to count many of my role models as my friends. This includes WBP co-founders Maria Teresa Ferretti and Antonella Santuccione Chadha who keep inspiring me. From them I learnt a lot about believing in yourself as well as never giving up on one’s dreams and daring to challenge current systems and propose new approaches.

What still needs to change for women in STEM?

The first step is surely in education. Many educational systems are considering an inclusive approach in their STEM curricula and this should be an essential element for all schools. It is also important to prioritize this more when allocating resources to our schools. We have many dedicated educators around the world but they also need the necessary resources and support to do their work. Accessibility to STEM subjects and programs of study should also be planned to include both young men and women providing diverse role models as well as offering adequate support for individual needs.

Last but not least, institutions and organizations in STEM should implement an inclusive and open approach in their policy to promote a culture that appeals to both men and women thus instilling an open and innovative approach while challenging traditional stereotypes.

Interview conducted by Associate Editor Karli Montague-Cardoso for World Mental Health Day & Ada Lovelace Day 2021

