# Author Correction: The Women’s Brain Project: an interview with Annemarie Schumacher Dimech on addressing gender in STEM and mental health research

**DOI:** 10.1038/s42003-021-02795-7

**Published:** 2021-10-27

Correction to: *Communications Biology* 10.1038/s42003-021-02699-6, published online 11 October 2021.

The original version of the article contained an error in the opening paragraph in which the ‘Women’s Brain Project (WBP) of Malta’ was referred to. The Women’s Brain Project is not affiliated with Malta. This has now been corrected in both the PDF and HTML versions of the Article.

